# Carotid Intima-Media Thickness, Genetic Risk, and Ischemic Stroke: A Family-Based Study in Rural China

**DOI:** 10.3390/ijerph18010119

**Published:** 2020-12-26

**Authors:** Mengying Wang, Siyue Wang, Xiaowen Wang, Junhui Wu, Yao Wu, Zijing Wang, Jiating Wang, Tao Wu, Yonghua Hu

**Affiliations:** Department of Epidemiology and Biostatistics, School of Public Health, Peking University, Beijing 100191, China; mywang@bjmu.edu.cn (M.W.); siyue.wang@pku.edu.cn (S.W.); wangxw@bjmu.edu.cn (X.W.); junhui@pku.edu.cn (J.W.); yaowu@pku.edu.cn (Y.W.); wangzijing14@pku.edu.cn (Z.W.); Jiating@pku.edu.cn (J.W.)

**Keywords:** carotid intima-media thickness, genetic risk, ischemic stroke

## Abstract

Background: Carotid intima-media thickness (cIMT) has been associated with an elevated risk of ischemic stroke (IS) in several studies, but the results are inconsistent. We investigated whether the association between cIMT and IS varied across different IS subtypes, and further assessed gene–cIMT interactions’ association with IS risk. Methods: A total of 1048 IS cases (795 large-artery atherosclerosis (LAA) cases, 103 small-vessel occlusion (SVO) cases, and 150 other subtypes) and 2696 IS-free controls across 2179 families were included in the analysis. Self-reported IS cases were confirmed through medical records’ review and head imaging by computed tomography and/or magnetic resonance imaging. The mean values of the common cIMT obtained in bilateral distal and proximal carotid artery segments were used. The genotype information of rs2910164 polymorphism in microRNA-146a (miR-146a) was also collected. Results: We found that cIMT was significantly associated with a higher risk of IS and LAA subtype but not SVO subtype in the multivariate-adjusted models. The odds ratio (OR) and 95% confidence interval (CI) in the highest quartile versus the lowest quartile of cIMT was 2.48 (1.92–3.20) for IS and 2.75 (2.08–3.64) for LAA (both *p* trend <0.001). The results also showed that there was a significant interaction between cIMT and rs2910164 genotype with the risk of IS (*p* interaction = 0.03) and LAA (*p* interaction = 0.02). The associations of cIMT with IS and LAA were strengthened among participants carried rs2910164_GG genotype compared with those with rs2910164_CC genotype. Conclusions: Our results indicate that higher cIMT levels were significantly associated with IS and LAA subtype but not SVO subtype, and the relations were modified by rs2910164 polymorphism in miR-146a.

## 1. Introduction

Stroke is a major contributor to disability-adjusted life years and death worldwide [1]. It is estimated that six million deaths are caused by stroke annually [2]. Notably, stroke is the leading cause of death, with 2.5 million new cases of the disease each year in China [3]. As the most prevalent subtype of stroke, ischemic stroke (IS) accounts for about 85% of all stroke cases [4]. In addition, most of IS cases are thromboembolic types, with atherosclerosis being the common cause [5]. Previous studies have indicated that the measurement of carotid intima-media thickness (cIMT) using B-mode ultrasound is an established marker for early atherosclerosis [6]. Moreover, several epidemiological studies have shown that cIMT may be associated with an increased risk of stroke [7,8,9], yet the results are inconsistent. Emerging evidence suggests that different IS subtypes might have various risk factors [2,10,11]. Therefore, we hypothesized that the discrepancies in the observed associations between cIMT and IS cross different studies might be partly due to different IS subtypes.

Furthermore, it is well recognized that stroke is a complex clinical syndrome resulting from both environmental and genetic factors [12]. Previous studies have shown that genetic susceptibility might interact with environmental factors regarding IS risk [13]. However, whether the association between cIMT and IS is modified by genetic factors has not been fully investigated. Compelling evidence has linked microRNAs (miRNAs) polymorphisms with the risk of IS. Notably, several studies have shown that the rs2910164 polymorphism in miR-146a is associated with IS [14,15,16]. Rs2910164 is located in the 3p pre-miR-146a region, and the variant could affect the expression of mature miR-146a [17,18]. Previous studies have indicated that miR-146a might be involved in the development of inflammation-related atherosclerosis through the regulation of interleukin-1 receptor-associated kinase 1 (IRAK-1), tumor necrosis factor receptor associated factor 6 (TRAF-6), Toll-like receptor, cytokine signaling pathway, nuclear factor-kappaB (NF-κB) pathway, MAP kinase pathway, and TNF-α expression, thus contributing to the pathogenesis of IS [19,20]. 

In the current study, we analyzed the association of cIMT with the risk of total IS and different IS subtypes in rural Chinese populations. We also investigated the interaction between miR-146a polymorphism and cIMT in the risk of IS.

## 2. Materials and Methods

### 2.1. Study Design and Participants

The present study was based on the baseline survey of an ongoing family-based cohort study in rural China. The protocol of the study has been described previously [21]. In brief, participants with IS were enrolled as probands from June 2005. Eligible parents and biological siblings of the probands were recruited by the proband-initiated contact method [22]. Self-reported IS cases were confirmed through medical records’ review and head imaging by computed tomography and/or magnetic resonance imaging according to the criteria of National Survey of Stroke [23,24]. In addition, IS cases were classified into five subtypes of large-artery atherosclerosis (LAA), small-vessel occlusion (SVO), cardioembolism, stroke of other determined etiology, and stroke of undetermined etiology based on the Trial of Org 10172 in acute stroke treatment (TOAST) [25]. The diagnosis and subtype confirmation of IS cases were conducted by two qualified neurologists from the department of neurology at Peking University Third Hospital. In order to preserve statistical power, only LAA (*n* = 795) and SVO (*n* = 103) subtypes were included in the subgroup analysis. Moreover, IS-free controls were defined as participants who were without self-reported stroke and responded negatively to all eight questions in the questionnaires for Verifying Stroke-Free Status (QVSFS) [26,27]. This study has been approved by the Peking University Institutional Review Board (IRB00001052-13027). All participants provided written informed consent.

After excluding participants with missing data on cIMT, 1048 IS cases and 2696 IS-free controls across 2179 families were included in the final analysis. Furthermore, 981 IS cases and 2547 IS-free controls with complete genotyping information were included in the gene-interaction analysis (Supplemental Figure S1).

### 2.2. Assessment of Carotid Atherosclerosis

The images of cIMT and carotid plaque were collected by two trained physicians from Fangshan District Center for Disease Control and Prevention using GE Vivid I ultrasound machine (GE Healthcare, Tokyo, Japan). The details have been described previously [28]. Briefly, the high-solution dynamic images of cIMT at three segments (proximal end, distal end, and bifurcation) were recorded for the far wall of carotid artery on each side, lasting for at least six cardiac cycles. In addition, the carotid plaques were scanned from the proximal end to bifurcation on both sides, with dynamic images in both coronal and sagittal plane recorded for each plaque.

Additionally, the measurement of cIMT was performed by trained staff using the Vascular Research Tools 6 DEMO software (Medical Imaging Applications LLC, Coralville, Iowa, USA) [28]. The cIMT was measured as the interval between two parallel bright lines on the image of the far wall of each segment at a position free of atherosclerotic plaque. The upper line represented the borderline between intima and lumen, while the lower line represented the borderline between media and adventitia. For each of the images, cIMT was measured twice at both systole and diastole and the averaged values were recorded. The type of cIMT measurement used in this study was the mean values of the common cIMT obtained in bilateral distal and proximal carotid artery segments.

The inter- and intra-class correlation coefficients for the two sonographers were ≥0.90. In addition, the inter- and intra-class correlation coefficients for the investigators who performed the measurements were 0.88 and 0.95, respectively.

### 2.3. Assessment of Covariates

The demographic information, lifestyle risk factors, and self-reported medical history were collected through face-to-face questionnaire survey by trained staff.

Physical measurements, including height, weight, and blood pressure were performed by trained investigators. A set of biomarkers including high-density lipoprotein cholesterol, low-density lipoprotein cholesterol, triglyceride, fasting blood glucose, and hemoglobin A1c were also collected. The body mass index (BMI) was calculated as weight (kg) divided by squared height (m^2^). Type 2 diabetes (T2D) was defined according to self-reported records or abnormal glycemic markers (fasting blood glucose ≥7.0 mmol/L or hemoglobin A1c ≥6.5%). Hypertension was defined as self-reported history, and/or systolic blood pressure ≥140 mm Hg, and/or diastolic blood pressure ≥90 mm Hg, and/or using anti-hypertensive medications. Prevalent coronary heart disease (CHD) was defined according to self-reported history of the disease. In addition, we collected the regular medication use of the participants, including lipid-lowering medications, anti-hypertensive medications, and hypoglycemic drugs.

### 2.4. Genotyping

DNA was extracted using LabTurbo 496-Standard System (TAIGEN Bioscience Corporation, Taiwan, China). In addition, the purity and concentration of DNA were measured using ultraviolet spectrophotometry [29]. Furthermore, the genomic DNA sample was genotyped with the time of flight mass spectrum using MassARRAY^®^ System (Agena Bioscience, San Diego, CA, USA). We used two negative (blanks) and three positive controls to control the quality of the genotyping process and the results were satisfied. We also chose 5% samples randomly for repeat analysis to verify reproducibility of the genotyping data.

### 2.5. Statistical Analysis

Categorical variables and continuous variables were described as percentages or median (interquartile range) according to baseline IS status. We used multilevel logistic regressions accommodating a family-based design to compare IS cases with IS-free controls for the variables. Multilevel logistic regressions accommodating a family-based design were used to calculate the odds ratio (OR) and 95% confidence interval (CI) for the association between cIMT and the risk of IS and subtypes using the lowest quartile of cIMT as the reference group. We adopted two multivariate models. In Model 1, we adjusted for age at recruitment (continuous) and sex (male, female). In model 2, we further adjusted for educational levels (less than primary school, primary school, middle school, high school, and college and above), alcohol consumption (current, former, never), smoking status (current, former, never), BMI (kg/m^2^, continuous), prevalent hypertension (yes/no), prevalent T2D (yes/no), and prevalent CHD (yes/no). If the covariate information was missing, we imputed mean values for continuous variables or used a missing indicator approach for categorical variables such as smoking status. The linear trend test was performed by treating cIMT as a continuous variable.

Based on priori hypotheses, we stratified the analysis by age (≤60 vs. >60 years), sex (male, female), BMI (≤28 vs. >28 kg/m^2^), hypertension status (yes/no), and T2D status (yes/no). We also conducted stratified analyses by rs2910164 genotype (CC, GC, and GG). The interaction test between cIMT levels and each category was performed by setting variable cross-product terms of cIMT with the variable in the models.

We conducted several sensitivity analyses to confirm the robustness of our results: further adjusted for high-density lipoprotein cholesterol (continuous) and triglyceride (continuous) in the model. Sensitivity analyses were also conducted by restricting to participants without self-reported CHD or lipid-lowering medication use at baseline for precise estimate on the observed associations.

All the statistical analyses were conducted using R version 3.6.3 (R Foundation for Statistical Computing, Vienna, Austria). *p*-values of less than 0.05 (two-sided) were considered statistically significant.

## 3. Results

The baseline characteristics of the participants according to IS status are shown in Table 1. Participants with IS were older, mainly males, and had lower education levels compared with those without IS. In addition, they were more likely to be current smokers. Besides, participants with IS had a higher prevalence of hypertension and CHD but a lower prevalence of T2D. We also observed that participants with IS had a lower rate of hypoglycemic drugs use than those without IS.

Table 2 shows the association between cIMT and IS. The mean (SD) of cIMT was 0.74 (0.15) and 0.68 (0.13) among IS cases and IS-free controls, respectively. We found that cIMT was significantly associated with a higher risk of IS in the age- and sex-adjusted, and multivariate-adjusted models. In the age and sex-adjusted model, a SD increase in cIMT was associated with 44% increased risk of IS (95% CI, 1.33–1.57). The OR (95% CI) was 3.02 (2.37–3.84) in the highest quartile versus the lowest quartile of cIMT (*p* trend *<* 0.001). After further adjustment for education levels, alcohol consumption, smoking status, BMI, diabetes, hypertension, and CHD, cIMT was associated with an increased risk of IS in a dose-response fashion. The OR (95% CI) of IS was 1.69 (1.33–2.16), 1.79 (1.41–2.28), and 2.48 (1.92–3.20), respectively in higher quartile groups compared with the lowest quartile group of cIMT (*p* trend *<* 0.001). 

In the subgroup analysis by IS subtypes, we observed that cIMT was significantly associated with LAA subtype (Table 2). In the multivariable-adjusted model, the OR (95% CI) of IS for a SD increase in cIMT was 1.42 (1.29–1.56). Compared with the lowest quartile of cIMT, the OR (95% CI) was 1.70 (1.28–2.24), 1.76 (1.34–2.32), and 2.75 (2.08–3.64) in higher quartile groups, respectively (*p* trend *<* 0.001) (Table 2). In addition, there was a significant association between cIMT and SVO in the age, sex-adjusted model (*p* trend = 0.01). However, the association disappeared after further adjustment for other covariates (*p* trend = 0.19).

We conducted stratified analyses by other potential risk factors. We did not observe significant interactions between cIMT and sex (male, female), obese status (BMI *<* 28 vs. BMI ≥ 28 kg/m^2^), diabetes (yes/no), or hypertension (yes/no) in relation to the risk of IS (Table 3). We found the association between cIMT and IS was consistent among these subgroups. However, we found a significant interaction between age and cIMT with the risk of IS (*p* interaction = 0.02), as the association between cIMT and IS appeared to be strengthened among participants with lower ages.

**Figure 1:**
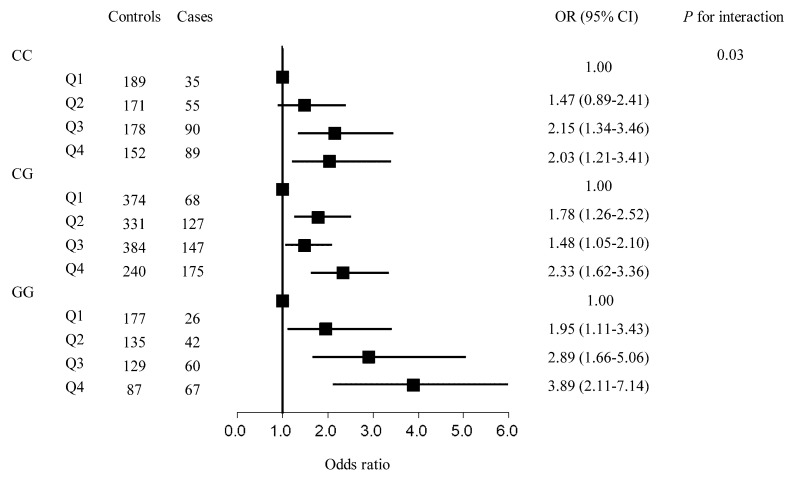
The association between carotid intima-media thickness (in quartiles) and ischemic stroke stratified by rs2910164 genotype. Results were adjusted for age (continuous), sex (male, female), educational levels (less than primary school, primary school, middle school, high school, college and above, missing), body mass index (continuous), alcohol consumption (current, former, never, missing), smoking status (current, former, never, missing), type 2 diabetes (yes/no), hypertension (yes/no), and coronary heart disease (yes, no, missing).

**Figure 2:**
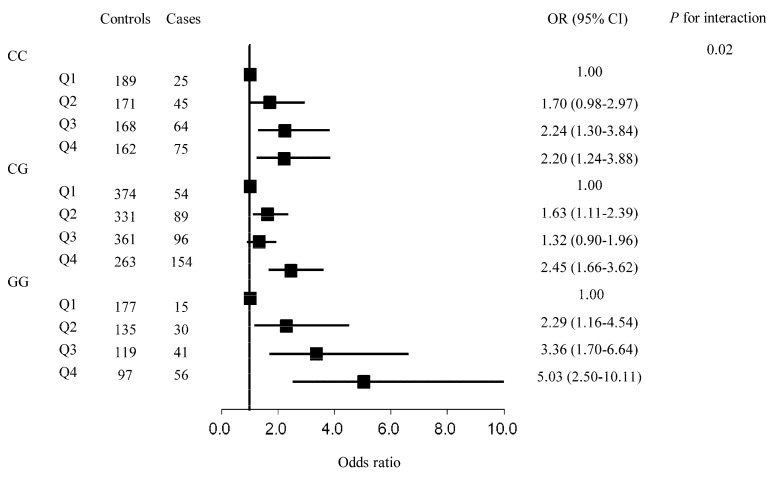
The association between carotid intima-media thickness (in quartiles) and large artery atherosclerosis ischemic stroke subtype stratified by rs2910164 genotype. Results were adjusted for age (continuous), sex (male, female), educational levels (less than primary school, primary school, middle school, high school, college and above, missing), body mass index (continuous), alcohol consumption (current, former, never, missing), smoking status (current, former, never, missing), type 2 diabetes (yes/no), hypertension (yes/no), and coronary heart disease (yes, no, missing).

We further performed stratified analyses by rs2910164 genotype. The results showed that there was a significant interaction between cIMT and rs2910164 genotype with the risk of IS (*p* interaction = 0.03) and LAA (*p* interaction = 0.02). Figure 1 shows the results of rs2910164-cIMT interaction for IS. Among participants who carried rs2910164_GG genotype, the OR (95% CI) of cIMT in the highest quartile vs. the lowest quartile was 3.89 (2.11–7.14) with the risk of IS; and the corresponding estimate was 2.03 (1.21–3.41) for participants with the rs2910164_CC genotype. In addition, the magnitude of rs2910164-cIMT interaction for LAA subtype is shown in Figure 2. We found that the OR (95% CI) of cIMT in the highest quartile vs. the lowest quartile with LAA subtype was 5.03 (2.50–10.11) among participants with rs2910164_GG genotype, which was higher than that of 2.20 (1.24–3.88) among participants with rs2910164_CC genotype.

The sensitivity analyses showed that the results were largely unchanged after further adjustment for high-density lipoprotein cholesterol and and triglyceride (Supplementary Table S1). In addition, after restricting to participants without self-reported CHD or lipid-lowering medications use at baseline, the results did not alter appreciably (Supplementary Table S2).

## 4. Discussion

In this family-based case-control study, we observed that a higher cIMT was significantly associated with increased risks of total IS and LAA subtype in a dose–response fashion, independent of other risk factors. However, there was no significant association between cIMT and SVO subtype. In addition, the associations were not modified by sex, BMI, hypertension, or diabetes. Moreover, the associations of cIMT with IS and LAA subtype were modified by rs2910164 polymorphism in miR-146a, as participants with s2910164_GG genotype had higher risks of IS and LAA subtype compared with the rs2910164_CC genotype.

A number of epidemiological studies have investigated the association between cIMT and IS, yet the results were inconsistently reported. The observed significant association between cIMT and IS in the present study was comparable with several prior investigations showing significant associations of cIMT with the risk of IS [7,30]. However, several studies did not find significant associations between common cIMT and IS [8,31]. Our results suggest that the discrepancies in previous observations might be at least partly due to different IS subtypes. Of note, we found that cIMT was associated with a higher risk of LAA subtype, but not for the SVO subtype, which was consistent with a previous investigation [7]. However, several studies reported significant associations between cIMT and lacunar strokes, although the associations were lower than LAA subtypes [32,33]. The discrepancy might arise from study design, sample size, and ethnic difference. In addition, previous studies have indicated that LAA and SVO might have a different pathogenesis, where SVO is commonly caused by the occlusion of small cerebral arteries due to lipohyalinosis and fibrinoid degeneration, but not atherosclerosis [34,35]. We assumed that this may partially explain the negative association in our analysis. Considering the limited number of SVO cases in our study, the results should be interpreted with caution.

Our findings of the significant association between cIMT and LAA subtype rather than SVO subtype is biologically plausible and of valuable clinical implications. Specifically, atherosclerosis is a progressive disease characterized by lipid-rich and inflammatory deposits in the sub-intimal space of arteries, which may lead to subsequent vascular endothelial dysfunction and plaque formation [36,37]. In particular, the rupture of atherosclerotic plaques in carotid arteries may lead to IS [38,39]. Large-artery atherosclerosis diseases are responsible for most of the IS cases, and atherosclerosis is the underlying process of the LAA subtype [5,40]. Notably, cIMT is a surrogate marker for the presence and progression of atherosclerosis [4], and the early detection of atherosclerosis using cIMT may help identify individuals at the risk for atherosclerotic IS [41]. Thus, our findings are of great importance for the prevention of IS, especially for LAA subtype. In addition, we did not find evidence for interactions by sex, BMI, hypertension, or T2D with cIMT in relation to the risk of IS in the stratified analyses. We observed a significant interaction between age, and cIMT with the risk of IS with the association was weakened among participants with higher ages.

Interestingly, we observed that there were statistically significant interactions between cIMT and rs2910164 genotype with the risk of IS and LAA subtype. The mechanisms by which interaction between rs2910164 polymorphism and cIMT might affect the risk of IS remain unclear. Recent evidence has revealed that the interactive impacts between genetic and environmental factors are partially regulated by epigenetic mechanisms [42]. In addition, several studies have shown that atherosclerosis is associated with some epigenetic changes including miR-146a gene polymorphism [42,43]. MiR-146 might be involved in the inflammation process through inhibiting the expression of IRAK-1 and TRAF6, which are key regulators downstream of Toll-like receptor and cytokine signaling pathways [44]. Furthermore, TRAF6 could regulate the expression of pro-inflammatory pathways including NF-κB pathway, MAP kinase pathway, and TNF-α [19,45]. As described in previous studies, endothelial inflammation plays a vital role in the development of atherosclerosis [46]. Thus, the down-regulation of miR-146a might contribute to the risk of IS and LAA subtype through the inflammation-related atherosclerosis process. In the study, we found that the association between cIMT and IS and LAA subtype appeared to be strengthened among participants with rs2910164_GG genotype compared with the CC genotype. Of note, C > G polymorphism in pre-miR-146a might down-regulate the expression of mature miR-146a [18,47], therefore providing feedback to induce excess inflammation, which plays an important role in the atherosclerosis and atherothrombotic stroke [45]. The findings from the current study are in line with prior evidence that genetic susceptibility may modify the relations between environmental factors and human health outcomes [48,49]. Our results may provide insights into the inconsistent observations on the relations between cIMT and IS in previous observational studies, in which the genetic modifications were not considered.

### Strengths and Limitations

Our study newly examined the associations between common cIMT and different IS subtypes, and provided novel evidence that the discrepancy of associations between cIMT and IS reported in previous investigations might be partly due to different IS subtypes. We also considered, for the first time, the modification effects of the genetic variations related to atherosclerosis in the association between cIMT and IS risk. Several potential limitations, however, should be addressed. First, although we controlled for demographic and lifestyle factors in the analyses, residual confounding could still be present. Second, a single measurement of cIMT was used in the study, which did not take into account the changes in intima-media thickness. Further studies with repeated measurements are warranted to assess the effect of changes in cIMT on IS. Third, we could not determine the causality of our findings due to the inherent limitation of the case-control study. Further longitudinal studies and clinical trials are warranted to assess whether the observed associations are causal. Fourth, the number of other IS subtypes cases was relatively small, somewhat limiting our ability to detect the association, if any, between cIMT and other IS subtypes. In addition, although we adopted imputation methods and missing indicator approaches to process missing variables in the current analysis, missing data might still reduce the statistical power and introduce potential bias. Finally, the participants in the genetic analysis were from rural China, which may limit the generalizability of the observed cIMT–gene interactions on IS risk to other populations.

## 5. Conclusions

In summary, our results indicate that increased cIMT levels are associated with high risks of IS and LAA subtype but not for the SVO subtype. Early detection of atherosclerosis using cIMT may be important for the prevention of the IS and LAA subtype. In addition, the associations are significantly modified by the rs2910164 polymorphism in miR-146a, as participants with rgw rs2910164_GG genotype may have a high risk of IS and LAA subtype related to cIMT.

## Figures and Tables

**Table 1:** Baseline characteristics of participants according to ischemic stroke status at baseline.

	Ischemic Stroke Cases	IS-Free Controls	*p*
	(*n* = 1048)	(*n* = 2696)	
Age (years)	61.0 (11.2)	58.0 (13.0)	*<*0.001
Male, %	61.7	47.4	*<*0.001
BMI (kg/m^2^)	26.2 (4.7)	26.1 (4.6)	0.37
Educational levels, %			*<*0.001
Primary school or less	48.6	38.4	
Middle school	39.7	44.5	
High school and above	10.9	16.4	
Smoking status, %			*<*0.001
Never smoker	43.4	55.2	
Past smoker	25.3	14.4	
Current smoker	30.5	29.9	
Alcohol drinking, %			0.73
Never drinker	56.9	60.9	
Past drinker	16.4	7.0	
Current drinker	25.6	31.5	
Hypertension, %	86.4	67.9	*<*0.001
Type 2 diabetes, %	36.3	40.9	0.01
Coronary heart disease, %	32.2	29.8	0.001
Antihypertensive treatment, %	63.5	45.3	0.93
Glucose lowering treatment, %	22.7	30.6	0.04
Lipid lowering treatment, %	25.0	13.0	0.72

Data are median (interquartile range) unless otherwise indicated. BMI, body mass index.

**Table 2:** Odds ratio and 95% confidence intervals of common carotid intima media thickness with ischemic stroke and its subtypes.

	Carotid Intima Media Thickness (mm, Quartiles)				Estimate of Per SD Increase	*p* for Trend
	Q1	Q2	Q3	Q4		
Ischemic stroke						
CIMT, mm ^a^	0.56 [0.52, 0.59]	0.64 [0.62, 0.66]	0.72 [0.70, 0.74]	0.85 [0.80, 0.92]		
Cases/control	137/772	235/665	314/743	362/516		
Model 1 ^b^	1.00	1.85 (1.46–2.34)	2.00 (1.58–2.52)	3.02 (2.37–3.84)	1.44 (1.33–1.57)	*<*0.001
Model 2 ^c^	1.00	1.69 (1.33–2.16)	1.79 (1.41–2.28)	2.48 (1.92–3.20)	1.36 (1.25–1.48)	*<*0.001
LAA						
CIMT, mm ^a^	0.56 [0.52, 0.58]	0.64 [0.62, 0.66]	0.71 [0.69, 0.74]	0.84 [0.80, 0.91]		
Cases/control	100/772	170/665	212/700	313/559		
Model 1 ^b^	1.00	1.82 (1.39–2.39)	1.92 (1.47–2.5)	3.24 (2.48–4.23)	1.50 (1.38–1.64)	*<*0.001
Model 2 ^c^	1.00	1.70 (1.28–2.24)	1.76 (1.34–2.32)	2.75 (2.08–3.64)	1.42 (1.29–1.56)	*<*0.001
SVO						
CIMT, mm ^a^	0.55 [0.51, 0.57]	0.63 [0.61, 0.65]	0.70 [0.68, 0.72]	0.81 [0.78, 0.88]		
Cases/control	11/661	19/689	36/666	37/680		
Model 1 ^b^	1.00	1.60 (0.75–3.39)	2.95 (1.46–5.92)	2.80 (1.36–5.75)	1.28 (1.06–1.54)	0.01
Model 2 ^c^	1.00	1.35 (0.63–2.89)	2.29 (1.12–4.66)	2.00 (0.95–4.20)	1.15 (0.93–1.40)	0.19

LAA, large artery atherosclerosis; SVO, small-vessel occlusion. ^a^ Data were expressed as median [25th, 75th percentile]. ^b^ Adjusted for age (continuous) and sex (male, female). ^c^ Adjusted for age (continuous), sex (male, female), educational levels (less than primary school, primary school, middle school, high school, college and above, missing), body mass index (continuous), alcohol consumption (current, former, never, missing), smoking status (current, former, never, missing), type 2 diabetes (yes/no), hypertension (yes/no), and coronary heart disease (yes, no, missing).

**Table 3:** Odds ratio and 95% confidence intervals of common carotid intima media thickness with ischemic stroke stratified by baseline risk factors.

	Carotid Intima Media Thickness (mm, Quartiles)				
	Q1	Q2	Q3	Q4	*p* for Interaction
Age ^a^					0.02
*<*60	1.00	1.75 (1.29-2.39)	1.83 (1.33–2.51)	3.28 (2.27–4.73)	
≥60	1.00	1.61 (1.07–2.43)	1.72 (1.17–2.51)	2.12 (1.45–3.09)	
Sex ^b^					0.76
Male	1.00	1.59 (1.13–2.24)	1.60 (1.15–2.23)	2.46 (1.74–3.46)	
Female	1.00	1.75 (1.23–2.49)	2.02 (1.42–2.87)	2.46 (1.66–3.64)	
BMI (kg/m^2^) ^c^					0.76
*<*28	1.00	1.70 (1.27–2.29)	1.77 (1.32–2.36)	2.59 (1.91–3.52)	
≥28	1.00	1.70 (1.10–2.64)	1.98 (1.28–3.04)	2.44 (1.53–3.90)	
Type 2 diabetes ^d^					0.16
No	1.00	1.44 (1.05–1.97)	1.65 (1.20–2.25)	2.20 (1.56–3.08)	
Yes	1.00	2.21 (1.50–3.26)	2.10 (1.43–3.07)	3.15 (2.11–4.69)	
Hypertension ^e^					0.34
No	1.00	1.68 (0.98–2.88)	1.14 (0.64–2.05)	1.60 (0.86–2.98)	
Yes	1.00	1.73 (1.31–2.28)	1.99 (1.52–2.61)	2.75 (2.07–3.65)	

BMI, body mass index. Variable list: educational levels (less than primary school, primary school, middle school, high school, college and above, missing), alcohol consumption (current, former, never, missing), and smoking status (current, former, never, missing). ^a^ Adjusted for sex (male, female), variable list, body mass index (continuous), type 2 diabetes (yes/no), hypertension (yes/no), and coronary heart disease (yes, no, missing). ^b^ Adjusted for age (continuous), variable list, body mass index (continuous), type 2 diabetes (yes/no), hypertension (yes/no), and coronary heart disease (yes, no, missing). ^c^ Adjusted for age (continuous), sex (male, female), variable list, type 2 diabetes (yes/no), hypertension (yes/no), and coronary heart disease (yes, no, missing). ^d^ Adjusted for age (continuous), sex (male, female), variable list, body mass index (continuous), hypertension (yes/no), and coronary heart disease (yes, no, missing). ^e^ Adjusted for age (continuous), sex (male, female), variable list, body mass index (continuous), type 2 diabetes (yes/no), and coronary heart disease (yes, no, missing).

## Data Availability

The data are not publicly available due to ethical issues.

## Supplementary Materials

The following are available online at https://www.mdpi.com/1660-4601/18/1/119/s1, Table S1: Odds ratio and 95% confidence intervals a of common carotid intima media thickness with ischemic stroke and its subtypes, Table S2: Odds ratio and 95% confidence intervals a of common carotid intima media thickness with ischemic stroke and its subtypes after limiting participants without self-reported CHD or lipid-lowering medications use, Figure S1: Selection of the participants and statistical analysis methods.
