# Supplementary material for: Carotid Intima-Media Thickness, Genetic Risk, and Ischemic Stroke: A Family-Based Study in Rural China
Source: Int J Environ Res Public Health. 2020 Dec 26;18(1):119. doi: 10.3390/ijerph18010119 (PMC7795493; doi:10.3390/ijerph18010119)
Supplement: Supplementary file 1 [file ijerph-18-00119-s001.pdf]

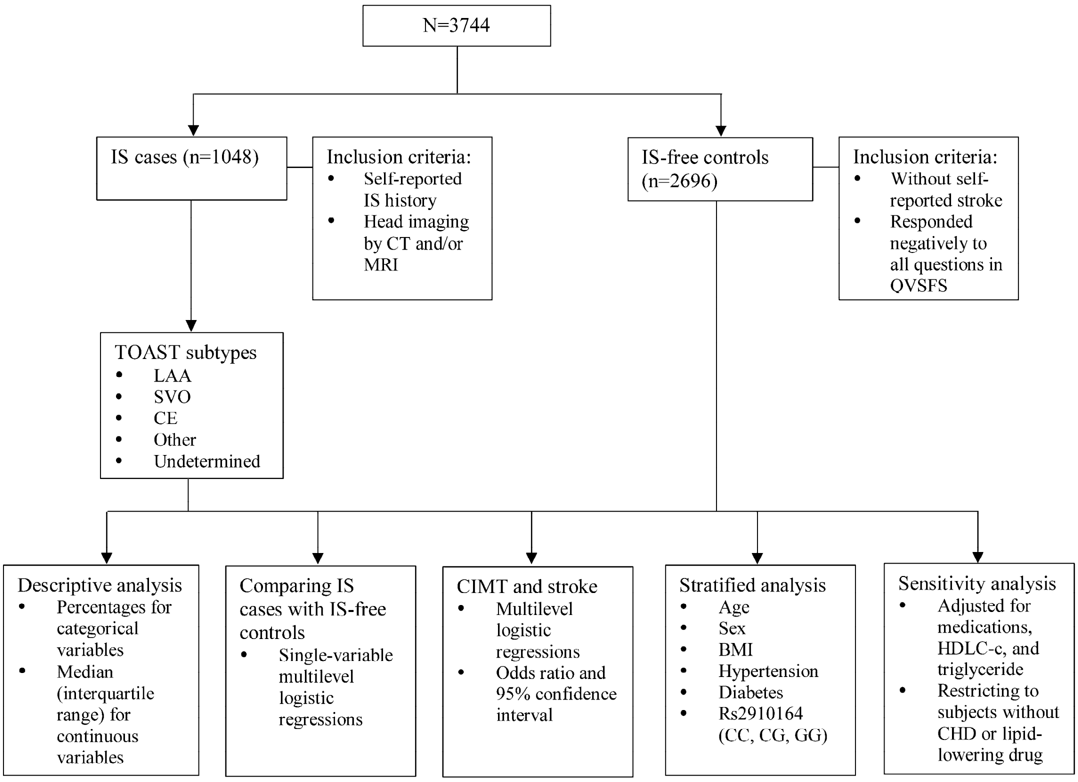

Supplementary Figure S1. Selection of the participants and statistical analysis methods.

Supplementary Table S1. Odds ratio and 95% confidence intervals [a] of common carotid intima media thickness with ischemic stroke and its subtypes.

	Carotid intima media thickness (mm, quartiles)				Estimate of per SD increase	*P* for trend
	Q1	Q2	Q3	Q4		
Ischemic stroke	1.00	1.70 (1.33-2.18)	1.81 (1.42-2.31)	2.55 (1.98-3.30)	1.37 (1.26-1.50)	<0.001
LAA	1.00	1.70 (1.29-2.25)	1.77 (1.35-2.33)	2.81 (2.12-3.71)	1.44 (1.31-1.58)	<0.001
SVO	1.00	1.39 (0.65-2.99)	2.36 (1.16-4.81)	2.08 (0.99-4.37)	1.16 (0.94-1.42)	0.16

LAA, large artery atherosclerosis; SVO, small-vessel occlusion. [a] Adjusted for age (continuous), sex (male, female), educational levels (less than primary school, primary school, middle school, high school, college and above, missing), body mass index (continuous), alcohol consumption (current, former, never, missing), smoking status (current, former, never, missing), type 2 diabetes (yes/no), hypertension (yes/no), coronary heart disease (yes, no, missing), high-density lipoprotein cholesterol (continuous), and triglyceride (continuous).

Supplementary Table S2. Odds ratio and 95% confidence intervals [a] of common carotid intima media thickness with ischemic stroke and its subtypes after restricting to participants without self-reported CHD or lipid-lowering medications use.

	Carotid intima media thickness (mm, quartiles)				Estimate of per SD increase	*P* for trend
	Q1	Q2	Q3	Q4		
Ischemic stroke	1.00	1.81 (1.26-2.59)	1.74 (1.21-2.50)	2.79 (1.87-4.14)	1.46 (1.27-1.69)	<0.001
LAA	1.00	1.89 (1.26-2.85)	1.90 (1.26-2.87)	3.02 (1.96-4.65)	1.53 (1.31-1.78)	<0.001
SVO	1.00	1.60 (0.50-5.10)	2.36 (0.73-7.60)	2.00 (0.54-7.49)	1.19 (0.80-1.78)	0.40

LAA, large artery atherosclerosis; SVO, small-vessel occlusion. [a] Adjusted for age (continuous), sex (male, female), educational levels (less than primary school, primary school, middle school, high school, college and above, missing), body mass index (continuous), alcohol consumption (current, former, never, missing), smoking status (current, former, never, missing), type 2 diabetes (yes/no), and hypertension (yes/no).
